# Stigma associated with early‐onset type 2 diabetes: A secondary qualitative analysis

**DOI:** 10.1111/bjhp.70042

**Published:** 2025-12-15

**Authors:** Jennifer Hagan, Melanie J. Davies, Jane Speight, Michelle Hadjiconstantinou

**Affiliations:** ^1^ Division of Global, Lifestyle and Metabolic Health, Diabetes Research Centre, College of Life Sciences University of Leicester Leicester UK; ^2^ Leicester Diabetes Centre University Leicester Hospitals Trust Leicester UK; ^3^ NIHR Leicester Biomedical Research Centre University Hospitals of Leicester NHS Trust and University of Leicester Leicester UK; ^4^ Australian Centre for Behavioural Research in Diabetes Diabetes Victoria Melbourne Victoria Australia

**Keywords:** early‐onset type 2 diabetes, psychosocial, qualitative, secondary analysis, stigma

## Abstract

**Objectives:**

Diabetes‐related stigma has been previously investigated in people with type 1 diabetes and older people with type 2 diabetes. However, stigma in early‐onset type 2 diabetes (EOT2D) remains understudied, despite increasing prevalence and the unique characteristics of this population. Hence, this study aimed to explore perceptions and experiences of stigma in people living with EOT2D, from the perspectives of people living with EOT2D and HCPs who have work in EOT2D care.

**Design:**

Secondary qualitative analysis of semi‐structured interviews.

**Methods:**

Semi‐structured interviews were conducted with 25 young adults with EOT2D and 25 HCPs. Data were analysed using inductive reflexive thematic analysis and subsequently presented using a framework for understanding diabetes‐related stigma.

**Results:**

Findings highlight causes, experiences, and consequences of stigma in EOT2D, in addition to potential mitigating strategies. The media, healthcare professionals and others perpetuate stigma, underpinned by misconceptions that type 2 diabetes is self‐inflicted, and attitudes of blame. PEOT2D experience judgement and harmful stereotypes, including ‘fat’, ‘unhealthy’, ‘lazy’, and type 2 diabetes being an ‘old‐person's disease’. Psychological consequences of stigma were reported, including self‐blame, embarrassment, and shame, leading to behavioural consequences of non‐disclosure and reluctance to seek help. Education, raising awareness, and non‐judgemental communication were highlighted as strategies to minimize stigma and its consequences.

**Conclusions:**

Stigma is highly pervasive in the lives of young adults living with EOT2D and can cause harmful consequences. Given the increased risk of physiological and psychological complications in this population, efforts to reduce stigma in EOT2D should be prioritized.

**Statement of Contribution**

**What is already known on the subject?**

- Diabetes‐related stigma is commonly experienced by people with diabetes and can lead to a range of psychological, behavioural and clinical consequences.
- There is a lack of evidence exploring diabetes‐related stigma in people living with early‐onset type 2 diabetes (EOT2D), despite their unique characteristics and increased risk of complications.

**What does this study add?**

- Both adults with EOT2D and HCPs report significant stigma in the lives of adults with EOT2D
- Adults with EOT2D are blamed for their condition by the media, HCPs, and others due to stereotypes
- Stigma in EOT2D causes feelings of embarrassment, self‐blame, and shame, leading to non‐disclosure

## INTRODUCTION

Type 2 diabetes (T2D) in younger adults (under 40 years old), known as early‐onset type 2 diabetes (EOT2D), is becoming increasingly common. In the UK, since 2016–2017, there has been a 40% increase in diagnoses of EOT2D, rising from approximately 120,000 people to nearly 168,000 people (Diabetes UK, 2024). Further, it is estimated that 50% of 16–44‐year‐olds with T2D are undiagnosed (Diabetes UK, 2024). Adults living with EOT2D are at a greater risk of severe complications, including microvascular and macrovascular complications (Nanayakkara et al., 2021; Soheilipour et al., 2023), some cancers (Yang et al., 2023), poorer pregnancy outcomes (TODAY, 2021) and mortality (Kaptoge et al., 2023). In addition to physiological consequences, adults living with EOT2D are more likely to experience mental ill‐health, such as depression, anxiety, and diabetes‐related distress (Barker et al., 2023; Browne et al., 2015; Liu et al., 2022). Diabetes‐related stigma is prevalent in this group and may play a role in these adverse consequences.

Diabetes‐related stigma refers to the negative attitudes and social judgements towards diabetes (Speight et al., 2024). Experiences of stigma in T2D have been explored within existing qualitative literature. People living with T2D report feeling blamed by others for causing their condition through lifestyle choices, experience negative stereotypes such as being lazy and obese, and face discrimination and loss of opportunities (Browne, Ventura, et al., 2013). Further, stigma presents various challenges on top of the complications already faced, and can have a negative impact on clinical, psychological and behavioural outcomes. For example, T2D stigma is associated with higher HbA1c, greater depressive symptoms and diabetes distress, higher frequency of complications, and reduced involvement in diabetes self‐management (Akyirem et al., 2023; Eitel et al., 2024). The causes, experiences, consequences, and mitigating strategies of T2D stigma have been summarized in a framework by Schabert et al. (2013).

Existing literature highlights the experiences and negative impact of diabetes‐related stigma on the wellbeing and self‐care of people living with T2D, although evidence is largely based on older samples (greater than 40 years old), meaning stigma in adults living with EOT2D remains understudied. Emerging evidence suggests adolescents and young adults with type 2 diabetes with higher levels of diabetes‐related stigma are more likely to use insulin, have higher HbA1c and experience chronic complications (Eitel et al., 2023), similar to older adults. However, literature suggests that age and time since diagnosis can influence experiences of diabetes stigma; for example, Zhang et al. (2023) found that a shorter diabetes duration was associated with greater diabetes stigma. Further, the experiences and impact of stigma on adults with EOT2D may differ from older individuals, due to the unique characteristics of the population (Browne, Scibilia, et al., 2013; Misra et al., 2022), such as managing diabetes while being of working age, being in full‐time education, or being of child‐bearing age. For example, existing qualitative research with adults living with EOT2D suggests that this group experiences stigma related to age‐specific factors, such as rejection from potential relationships, unfair negative assessment at work, and judgement for having diabetes (which is often perceived as an older person's condition) at a younger age (Croke et al., 2023; Wong et al., 2024).

Further exploring stigma in this population and examining the potential role of age would provide valuable insights that could improve understanding of their experiences and care needs.

Therefore, the aim of this study was to explore perceptions and experiences of stigma in EOT2D, from the perspectives of adults living with EOT2D and healthcare professionals (HCPs). Exploring the views of both groups offers insight into diabetes stigma from those who experience it and from those who might unintentionally contribute to it. This provides a novel perspective of diabetes stigma, which remains underexplored in current literature.

## METHODS

This paper describes a secondary analysis of interview data from a qualitative study which aimed to explore and understand the unmet needs of adults living with EOT2D and their diabetes care within England (Chauhan et al., 2025). In the original study, semi‐structured interviews were conducted with adults living with EOT2D and HCPs, and an abductive approach to analysis was taken, using the normalization process theory to analyse and interpret the data. During the primary analysis of this dataset, stigma was identified as a recurring theme, yet it could not be captured effectively within the scope of the primary analysis. Thus, we aimed to conduct a secondary analysis to explore this area in greater detail.

Research Ethics Committee (REC) favourable opinion and HRA approval were obtained from the East Midlands, Nottingham 1 REC (22/EM/0014).

### Participants

All individuals who expressed interest in participating were provided with a study invitation pack. Two versions of the pack were developed: one for adults with EOT2D and one for healthcare professionals (HCPs). Each pack included a reply slip containing sociodemographic questions. All study materials were reviewed and informed by input from patient and public involvement members.

#### Adults with EOT2D

Adults with EOT2D aged 16 to 40 years (inclusive) were recruited to take part in this study. Recruitment took place via General Practices (GP) and specialist hospital services across the East Midlands and North‐West London, including: the University Hospitals of Leicester NHS Trust (UHL), Nottingham University Hospitals NHS Trust, University Hospitals of Derby and Burton (UHDB) and Imperial College Healthcare NHS Trust. Advertising on social media and ‘word of mouth’ were also used. Further details on recruitment are explained in the primary manuscript (Chauhan et al., 2025).

#### HCPs

HCPs aged 18 to 75 years (inclusive) who had been and/or were currently in contact with people with T2D were invited. Recruitment avenues included GP and hospital specialist services, social media, poster distribution at national and local training events and meetings, and existing interprofessional networks.

Informed consent was obtained prior to participation in the primary study. Within the consent form, all participants (adults with EOT2D and HCPs) confirmed that interview data may be analysed further.

### Data collection

Semi‐structured interviews were conducted between July 2022 and May 2023. Two topic guides were developed: one for adults living with EOT2D and one for HCPs. Topic guides were mapped onto the constructs of the Capability‐Opportunity‐Motivation‐Behaviour (COM‐B) model (Michie et al., 2011) to explore individual capacity, opportunity and motivational factors that could impact daily self‐management behaviours in adults with EOT2D. Topic guide questions were also mapped onto relevant constructs of the normalization process theory (May et al., 2022) to facilitate identification of factors that might influence the implementation of EOT2D care at an individual and collective level.

Interviews were facilitated by a qualitative researcher with a background in Psychological Well‐being. The first interviews were co‐facilitated by a senior qualitative researcher (MH). Interviews took place via telephone, Microsoft Teams, or face‐to‐face depending on participant preference. The interviews were audio recorded and transcribed verbatim by an external professional transcription service.

### Data analysis

NVivo 12 qualitative software was used to facilitate data management and analysis. Data were reanalysed using reflexive thematic analysis (Braun & Clarke, 2019, 2024). Data were coded by a qualitative researcher with a background in health psychology and experience conducting thematic analysis (JH). Throughout, coding was critically discussed with a senior qualitative researcher (MH) to facilitate reflexivity. To ensure familiarization with the data, JH repeatedly read through the data and the field notes. Further, JH developed their understanding of EOT2D through literature reading.

A constructionist approach to analysis was undertaken, which seeks to understand the social contexts influencing an individual's account of meaning and experiences (Braun & Clarke, 2006). The analysis was inductive; themes were generated exclusively from the data, not based on the researcher's preconceptions. Meaningful codes were organized into themes, then iteratively refined. Coding took place at both a semantic and latent level, capturing explicit and implicit meanings of the data. Following the inductive analysis, the authors noted that the findings aligned closely with Schabert et al. (2013)'s framework for understanding diabetes stigma (Schabert et al., 2013). Hence, the framework was then used to facilitate the presentation of the data, encompassing sources, experiences and consequences of stigma.

## RESULTS

In total, 50 participants were interviewed (25 adults living with EOT2D; 25 HCPs). Interviews with adults with EOT2D ranged between 10 and 81 min, whilst interviews with HCPs ranged between 52 and 73 min.

Of the 50 transcripts analysed, 18 had no data relevant to stigma; 9 HCPs (36%) and 9 adults living with EOT2D (36%). Hence, 32 participants were included in the secondary analysis. The majority of adults with EOT2D who discussed stigma were female (87.5%) compared to those who did not discuss stigma (54%), but were a similar age (87% aged 25–39 and 88% aged 25–39 respectively).

Of the adults living with EOT2D, the majority were from a white background (*n* = 12, 75%), aged between 25 and 39 years (*n* = 14, 87.5%), and had been diagnosed with T2D in the last 3 to 10 years (*n* = 11, 68.8%).

Of the HCPs, the majority were female (*n* = 9, 56.3%), from a white background (*n* = 12, 75%), aged between 40 and 59 years (*n* = 12, 75%), and working as specialist care providers (SCPs) (*n* = 15, 93.7%). Participant demographic characteristics are presented in Tables 1 and 2.

**TABLE 1:** Demographic characteristics of adults living with EOT2D.

	Total (*n* = 18)	
	*n*	%
*Gender*		
Male	2	12.5
Female	14	87.5
Non‐Binary	0	0
Not listed	0	0
Prefer not to say	0	0
*Age*		
16–24 years	0	0
25–39 years	14	87.5
40 years	2	12.5
*Ethnicity*		
Asian or Asian British	4	25
Black or Black British	0	0
White	12	75
Mixed or multiple ethnic group	0	0
Another ethnic group	0	0
Other	0	0
*Length of diabetes diagnosis*		
Less than 6 months	1	6.2
Between 1 and 3 years	3	18.8
Between 3 and 10 years	11	68.8
10+	1	6.2
*Education*		
Secondary school	2	12.5
Sixth form/College	5	31.3
Undergraduate degree	6	37.5
Postgraduate degree	2	12.5
Prefer not to say	0	0
Missing	1	6.2
*Confidence using internet resources*		
Very confident	11	68.8
Confident	4	25
Slightly confident	1	6.2
Not confident	0	0
Missing	0	0
*Number of long‐term health conditions*		
1	5	31.3
2	4	25
3	4	25
4 or more Missing	3 0	18.7

**TABLE 2:** HCP demographic characteristics.

	Total (*n* = 16)	
	*n*	%
*Gender*		
Male	7	43.7
Female	9	56.3
Non‐binary	0	0
Not listed	0	0
Prefer not to say	0	0
*Age*		
18–24 years	0	0
25–39 years	3	18.7
40–59 years	12	75
60–75 years	0	0
Missing	1	6.3
*Ethnicity*		
Asian or Asian British	2	12.5
Black or Black British	1	6.3
White	12	75
Mixed or multiple ethnic group	0	0
Another ethnic group	0	0
Other	1	6.3
*Role in type 2 diabetes care* ^a^		
Dietitian	3	18.7
Diabetes specialist nurse	5	31.2
General practitioner	1	6.2
Diabetes consultant	4	25
Healthcare assistant	0	0
Other (i.e. Registrar, SMES^b^ educators)	3	18.7
Missing	1	6.2
*Years of experience in role*		
6–12 months	0	0
1–5 years	2	12.5
5–10 years	8	50
10+ years	5	31.2
Missing	1	6.3
*Years of experience treating young adults with type 2 diabetes*		
None	0	0
Less than 6 months	0	0
6–12 months	0	0
1–5 years	1	6.3
5–10 years	7	43.7
10+ years	7	43.7
Missing	1	6.3
*Type of healthcare institution* ^a^		
Primary	3	18.7
Secondary	10	62.5
Community	4	25
Missing	1	6.3

^a^ Selected more than one answer.

^b^ Self‐management education and support.

The themes in the current secondary analysis were generated from 32 transcripts: 16 HCPs and 16 adults living with EOT2D.

**FIGURE 1:**
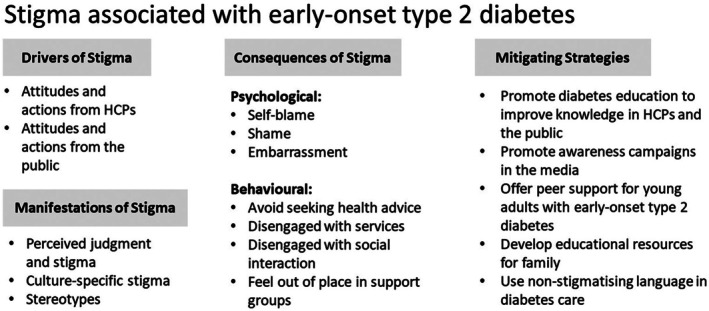
Summary of stigma associated with early‐onset type 2 diabetes.

Codes were mapped onto four overarching themes, in line with the Schabert et al. (2013) framework for understanding diabetes stigma: (i) drivers of stigma, (ii) experiences of stigma, (iii) consequences of stigma, and (iv) mitigating strategies. These themes encompass 9 further subthemes (see Figure 1).

### Drivers of stigma

Both groups identified various factors that can contribute to the development of stigma in EOT2D, which is reinforced by sources including HCPs, the public and the media.

#### Sources

The attitudes and actions of HCPs can contribute to diabetes‐related stigma. Both groups described the judgemental attitudes of many HCPs, which impose blame and shame. HCPs ‘telling off’ individuals for their management was also reported. Some felt that HCPs lack awareness of the complex risk factors for T2D and that this could be a reason for their stigmatizing attitudes/behaviours.

…there's a lot of flippant attitudes, even amongst HCPs… You're overweight, you just need to lose weight, things like that so yeah, people don't realise the impact psychologically on those patients. (HC06, Female, 40–59 years, Diabetes Specialist Nurse (DSN))

Both groups also described an overarching perception of the public that T2D is the fault of the individual. They viewed the public as lacking understanding of T2D. Participants felt that these misconceptions reinforce the belief that T2D is not seen as acceptable in society, amplifying stigmatizing attitudes and behaviours.

…they (the public) automatically think of like massive Americans, that they don't have an understanding of it themselves. So, they kind of like just jump on the bandwagon kind of thing and it's like we're not all like that. (P022, Female, 25–39 years)

The media was also highlighted as a key source of T2D stigma. Both groups noted the media portrays T2D negatively, spreading messaging that T2D is self‐inflicted, and that people living with T2D ‘waste healthcare resources’. The misconception that T2D is an older person's condition is also frequently depicted in the media.

Any time that anything to do with diabetes is reported in the news, it's always fat people this, fat people that. Strain on our society, we shouldn't be providing healthcare and prescriptions to them for free, it's their own fault, they need to stop eating. (P008, Female, 25–39)

#### Features of the condition

Various features of T2D can contribute to the development of stigma. Both groups discussed self‐management behaviours as a potential source of stigma, particularly when enacted in public. For example, injecting insulin, taking medication, attending clinic appointments and going to the gym.

If say for example, they're on insulin, it's difficult taking their medication. There's a bit of stigma surrounding that. (HC22, Female, 40–59 years, DSN)

Pre‐existing weight stigma can lead to further stigma in EOT2D through generalizations. Adults with EOT2D described significant weight stigma in society, resulting in assumptions of unhealthy lifestyle choices. A strong focus on weight in healthcare consultations was also noted.

Being a larger person and having T2D you basically are told a numerous amount of times “You've done it to yourself” and with that then seems to go, I don't know, a little bit of lapse in care of actually caring for you in a way. (P019, Female, 25–39 years)

#### Intersecting stigma

The above features of diabetes can contribute to the psychosocial mechanisms driving stigma. Due to the negative connotations associated with overweight, both groups noted that the public and HCPs develop the perception that EOT2D is self‐inflicted, and blame adults for their own condition.

There's still quite a lot of stigma for obesity and having T2D at a young age because of your obesity, I believe for young people is quite, carries quite a significant stigma because then it is all associated with the fact that you have been eating more… you became fat so that's why you got diabetes. (HC18, Female, 40–59 years, Diabetes Consultant)

### Manifestations of stigma

Due to the previously outlined causes and sources of stigma, adults living with EOT2D describe experiencing stigma in their everyday lives. Sources of stigma enact it through behaviours and judgement. This reinforces stereotypes relating to blame and shame.

The perceived stigma and stereotypes experienced by participants are reported below.

#### Perceived stigma

Perceived judgement from others was highlighted; adults living with EOT2D experienced unsolicited, judgmental advice, for example, suggestions of lifestyle changes.

When I was first diagnosed, I did get a lot of “Oh you're too young to have diabetes”. Maybe try changing this. And I think to myself “If I wanted your advice, I'd ask for it”. (P025, Female, 25–39 years)

Adults living with EOT2D also felt that others judged their lifestyle habits due to preconceived ideas about T2D; others assume they are making unhealthy choices purposefully and do not consider individual circumstances, such as the affordability of healthy food. One participant described culture‐specific beliefs, which result in judgements about their personal lives.

In my culture…they correlate diabetes with being not relationship‐, marriage‐material, or can't have kids, that's how they perceive it. (P009, Female, 25‐39).

#### Stereotypes

Both groups reported that negative stereotypes are imposed, for example, that people with EOT2D are unhealthy. They reported that adults with T2D, generally (i.e. not just adults with EOT2D), are perceived to be greedy, eat unhealthy foods, and in large quantities:

Basically, he [GP] even said the words to me ‘sat on the sofa eating Tate & Lyle’, meaning sugar, putting sugar down your face. I don't even like sweet things. (P021, Female, 25–39 years)

Further, participants described portrayals and descriptions of people with EOT2D frequently depicting overweight individuals. Participants also described that it is often assumed that no‐one with EOT2D exercises and that they live sedentary lives which causes their T2D.

So, whenever they talk about T2D there's always a person in a large body, sat on a bench, headless, but with some pies or some crisps or something alongside, and that's not the case. (HC12, Female, 40–59 years, DSN)

It was felt that many individuals believe T2D is diagnosed in older groups. Some adults living with EOT2D reported being mislabelled as having T1D because of their younger age, and being told that they are ‘too young’ to have diabetes.

You kind of expect it [T2D] for people in old age. But for younger people, it's seen as—you've let your life get out of control and you're a burden on society and you're fat and ugly and whatnot (P002, Female, 25–39 years)

As a result of the above stereotypes, participants described an overarching stereotype that T2D is self‐inflicted. Many people believe that T2D is caused by the individual, through poor lifestyle behaviours and choices.

I think heart disease, diabetes, stroke all have this slight stigma attached to them that, you know, you've not been healthy, you've not been exercising, you've not been eating well, you've been smoking, you've been drinking. And if you do those things or if you've ever done those things, you're bound to pick up one of these diseases and each were in fault. (HC21, Male, 25–39 years, General Practitioner)

### Consequences of stigma

As a result of the aforementioned experiences of stigma, participants described the psychological and behavioural consequences that can arise. Adults living with EOT2D reported feeling embarrassed, ashamed and blaming themselves for their condition. These adverse feelings can then lead to behavioural consequences: feeling too embarrassed to talk about their diabetes with others, resulting in non‐disclosure and reduced help‐seeking behaviours.

The psychological and behavioural consequences of stigma, as reported by participants, are described in further detail below, with illustrative quotes.

#### Psychological

Diabetes stigma was reported to have a range of negative psychological consequences, feeling embarrassed about having T2D, or feeling like a failure. They did not want to be seen as having T2D, which makes engaging in self‐management behaviours in public uncomfortable. They also reported feeling embarrassed talking about their diabetes with others.

I mean it's still sort of embarrassing to talk about you know, because if you admit you've got T2D, then it's almost like you're admitting you're a failure, you know? (P002, Female, 25–39 years)

Adults living with EOT2D also reported blaming their own lifestyle choices and felt at fault due to the stereotypes that imply this. They described feeling isolated with some describing blaming themselves for not taking care of their health, and for impacting their family members' quality of life.

I felt it (EOT2D) was completely my fault for the lifestyle choices that I had made to that point…. (P004, Female, 25–39 years)

I feel like it can be quite isolating to find out you're diabetic, and as a younger person, there's a lot of shame involved. (P008, Female, 25–39 years)

#### Behavioural

In their experience of working with adults living with EOT2D, HCPs described reduced engagement from adults with EOT2D with healthcare services due to stigma. HCPs reported individuals fearing being told off and judged, which can arise from previous negative experiences with other HCPs. HCPs also felt that individuals refuse to attend T2D support groups, as they do not want to be labelled with the associated stereotypes. They also described individuals feeling out of place in clinics, due to the association of T2D with older people.

I've spoken to one young person who wasn't coming to her clinic appointments because she didn't want to be sat in the waiting room with other people who she regarded as not being like her, like older people with sort of bandaged leg or you know, I've got nothing in common with any of these people and I don't want to be associated (HC14, Female, 40–59 years, DSN)

### Strategies to reduce diabetes stigma

Participants identified strategies to mitigate stigma which could target causes, experiences and consequences of stigma. Improving knowledge/awareness could consequently reduce attitudes of blame and subsequent stereotypes. Social support can help reduce psychological consequences, such as feelings of embarrassment, shame and self‐blame.

These strategies, as identified by participants, are detailed further below.

#### Education

Improving knowledge and awareness of diabetes within the general public and HCPs was highlighted as a potential strategy to reduce diabetes stigma. In particular, both groups felt there was a lack of awareness that T2D is not always due to lifestyle choices. They described a need to raise awareness about this, which they felt would help destigmatize T2D.

I think raising the awareness is an important thing. And specialist teams, specialist community teams know that anybody can develop T2D; it's not necessarily something that somebody's brought on themselves. But I wonder sometimes whether that is not known within the general population, perhaps within general practice (HC12, Female, 40–59 years, DSN)

Awareness campaigns in the media were suggested to achieve this. Providing educational resources to friends and family members was also suggested by adults with EOT2D, who thought this would help to share important information, and normalize T2D.

If we give them resources to take home to share with their family and friends so that they know how to support them and not look at them like they're weird, or not look at them because they're having to prick their finger at the dinner table, I think that would help… (P008, Female, 25–39 years)

#### Social support

Participants also felt that social support can be helpful in reducing stigma. HCPs were aware that many adults living with EOT2D fear blame, and being ‘told off’; therefore, HCPs reported taking care when speaking to them, ensuring they use language that is not accusatory.

Our ethos in the young adult clinic anyway is one of “No blame, no shame”—you're not going to get given a hard time. You're not going to be told off and humiliated or any of those things. (HC09, Female, 40–59, Dietitian)

The benefits of peer support in relation to stigma was also discussed. Adults living with EOT2D felt that meeting and speaking with others like them helps reduce embarrassment.

Eventually you can end up going to one of these meetings, and it can be the difference between being embarrassed and ashamed of what's happening to you, between feeling completely normal and knowing that actually, there's so many people in the world like you. (P008, Female, 25–39 years)

## DISCUSSION

This secondary analysis is the first study to explore the perceptions and experiences of stigma associated with EOT2D, including the perspectives of adults living with EOT2D and HCPs. Findings broadly aligned with Schabert et al.'s (2013) framework for diabetes stigma, which was used to present the data, providing an overview of the sources, experiences, and consequences of diabetes stigma in an EOT2D population, in addition to possible mitigating strategies.

Stereotypes were identified as common experiences of stigma. Consistent with existing findings (Browne et al., 2014; Browne, Ventura, et al., 2013), participants explained that T2D is portrayed as a self‐inflicted disease, labelling people living with EOT2D as fat and unhealthy. In addition to the potential psychological consequences of these stereotypes, they could also lead to a lapse in care. Physicians reported being repulsed by adults with T2D, viewing them as lazy and non‐compliant (Bennett & Puhl, 2023). This reinforces the need for HCP training and interventions to reduce stigma, and improve communication skills. As noted by participants in the current study, non‐judgemental language from HCPs can help to minimize feelings of blame and reduce stigma and its consequences.

In addition to this stereotype being inflicted by HCPs, participants in the current study noted similar judgements from the general population. This reinforces existing literature that highlights peer‐based stigma in younger age groups. For example, a recent study with university students found that this age group expressed negative attitudes and stereotypes towards people with diabetes, with 30% agreeing that people with diabetes are responsible for their condition (Khalafalla et al., 2024). Given the significance of peer relationships in this age group (Eitel et al., 2024), perceptions of stigma in EOT2D may be exacerbated by this.

Another key stereotype highlighted in the current study is that T2D is seen as an ‘old person's condition’, despite the significant rise in EOT2D cases (Savage et al., 2009). This finding echoes existing qualitative research with adults living with EOT2D, who have reported feeling embarrassed due to having diabetes at a young age, and have been told they are too young to have the condition (Croke et al., 2023; Wong et al., 2024). Existing literature has also revealed that adults living with EOT2D feel that T2D information, resources, and support are heavily tailored towards older individuals (Rasmussen et al., 2016; Savage et al., 2009), which is perhaps a result of this persistent stereotype.

The psychological and behavioural consequences of stigma described by participants echo existing quantitative and qualitative findings from T1D and older T2D populations. For example, feelings of shame, self‐blame, and embarrassment, lead to non‐disclosure and a reluctance to seek help (Akyirem et al., 2023; Browne, Ventura, et al., 2013); the current study provides novel evidence of these findings in an EOT2D population. Existing research has also identified an association between stigma and sub‐optimal clinical outcomes in this population, including higher HbA1c and an increase in complications (Eitel et al., 2023, 2024). While this could not be evidenced in the current analysis, the identified behavioural consequences of stigma could have a subsequent negative clinical impact.

The negative psychological impact of stigma revealed in the current study underscores the need for psychological support in EOT2D care, which has been previously identified, considering the increased risk of psychological complications in this population. Multi‐disciplinary approaches to EOT2D care that encompass psychosocial support, such as the NHS England Type 2 Diabetes in the Young (T2Day) programme (NHS, 2023), should therefore be encouraged (Misra et al., 2023). Effectively targeting stigma and psychological well‐being could have an additional positive impact on the identified behavioural consequences of stigma, given that self‐stigma and poor mental health are associated with a reduction in self‐management (Kato et al., 2020; Schmitt et al., 2021).

### Strengths and limitations

We acknowledge the limitations that often appear with secondary qualitative analysis. However, secondary data analysis can be a credible research methodology, and the four criteria for methodological rigour were considered (Korstjens & Moser, 2018): to ensure *credibility*, researchers from the original qualitative study team were involved in the interpretation of the secondary analysis data, and two researchers held regular meetings during the data analysis process to support the qualitative researcher's reflexivity and interpretation of the data. To strengthen *transferability*, we have presented detailed information on the data collection and analysis process, including the setting and characteristics of the participants. Moreover, we have summarized and referenced the primary qualitative study which features a detailed description of the study's methods. To enhance *confirmability* and *dependability*, we followed core principles of RTA, acknowledging our positionality and interpretation of the findings.

This study has several strengths. Clean, uncoded transcripts were used to ensure data were analysed from a new perspective. Additionally, by analysing interviews with HCPs, we were able to gain insights into EOT2D stigma from two perspectives. Such perspectives are yet to be explored in existing literature; therefore, the novel data obtained is a key strength of the current study.

The majority of adults living with EOT2D in the current study were female (76%); existing literature suggests that diabetes stigma is higher in females compared to males (Eitel et al., 2024; Pedrero et al., 2021; Taher et al., 2023); therefore, the current sample may be overrepresented with individuals experiencing stigma. Qualitative research with a greater sample of males would help determine this, which might identify any gender‐related differences in experiences and perceptions of diabetes stigma.

Further, despite the ethnic diversity of recruitment strategies, the sample of adults with EOT2D was majority white (64%), with no individuals from a Black ethnic group. Given the high rates of EOT2D in ethnic minority groups, it is vital that their views are represented. This is particularly important in consideration of existing literature highlighting the impact of culture on diabetes stigma (Goff et al., 2020). For example, in some Asian communities, diabetes is seen as a sign of physical inadequacy and can impact marriage prospects (Abdoli et al., 2018), thus exploring culture‐related stigma in minority ethnic groups with EOT2D would be beneficial.

Finally, the topic guides for the primary study were developed with regard to an aim not directly related to stigma and it is likely that not all aspects of stigma were explored. For example, some components of Schabert et al. (2013)'s framework, including stigmatizing practices (such as reductions in work opportunities) and medical consequences, were not discussed. Therefore, further qualitative research aiming to explore stigma in EOT2D from the outset, with topic guides developed for this aim, could fill gaps identified by the current analysis. However, despite questions not directly addressing stigma, it was evident throughout the majority of the interviews highlighting the strong presence of stigma in EOT2D.

### Implications

Our findings have real‐world implications. The consequences of stigma revealed highlight the importance of tackling diabetes stigma. The findings suggest that stigma can have a negative impact on mental health and lead to reduced engagement in healthcare and self‐management. Thus, it is vital that efforts are made to minimize these consequences to prevent further adverse effects.

Raising awareness and improving knowledge of EOT2D in the general public and in HCPs was identified as a potential strategy to minimize stigma. The use of non‐stigmatizing language to minimize blame and shame should be emphasized in HCP training, with the promotion of resources such as NHS England's ‘Language Matters’ guide (Cooper et al., 2018).

## CONCLUSIONS

This secondary analysis highlights the complex nature of stigma in EOT2D. Stigma can arise due to attitudes of blame, and is perpetrated by the public, HCPs and the media. Adults living with EOT2D experience stigma in the form of harmful stereotypes and harsh judgements, which can lead to a range of psychological and subsequent behavioural consequences. Mitigating stigma through strategies aiming to improve awareness and knowledge of EOT2D, and encourage non‐judgemental language in HCPs, should therefore be a priority.

## AUTHOR CONTRIBUTIONS

**Jennifer Hagan:** Formal analysis; writing – original draft. **Melanie J. Davies:** Writing – review and editing; funding acquisition; supervision. **Jane Speight:** Writing – review and editing. **Michelle Hadjiconstantinou:** Conceptualization; investigation; methodology; formal analysis; supervision; writing – original draft; writing – review and editing.

## FUNDING INFORMATION

This study is funded by the National Institute for Health and Care Research (NIHR) under its Programme Grants for Applied Research Programme (NIHR201165) and by NOVO Nordisk. The views expressed are those of the author(s) and not necessarily those of the NIHR or the Department of Health and Social Care.

## ETHICAL APPROVAL

Research Ethics Committee (REC) favourable opinion and HRA approval was obtained from the East Midlands, Nottingham 1 REC (22/EM/0014). Informed consent was obtained from all participants prior to their participation in the study.

## Data Availability

The data that support the findings of this study are not openly available due to reasons of sensitivity and are available from the corresponding author upon reasonable request. Data are located in controlled access data storage at the University of Leicester.
